# Echocardiographic guided, transatrial closure of a patent foramen ovale

**DOI:** 10.1186/s13019-020-01289-7

**Published:** 2020-09-14

**Authors:** Felix Fleissner, Paul Frank, Axel Haverich, Issam Ismail

**Affiliations:** 1 Division of Cardiac, Thoracic, Transplantation and Vascular Surgery, Hannover Medical School, Carl-Neuberg- Strasse 1, 30625 Hannover, Germany; 2 Clinic for Anesthesia and Critical Care Medicine, Hannover Medical School, Hannover, Germany

**Keywords:** Patent foramen ovale, PFO, Off-pump-surgery, OPCAB, CABG, Valve surgery

## Abstract

**Background:**

The management of an incidental patent foramen ovale found during planned cardiac surgery remains a challenge, and current guidelines are not helpful. Although evidence is accumulating, that closure of an incidental found patent foramen ovale might be beneficial, especially in planned off-pump procedures, the diagnosis of a formerly unknown patent foramen ovale with the patient on the operation table has vast consequences by making it necessary to switch to on pump, bi-caval cannulation for patent foramen ovale closure. We therefore developed a technique for transatrial closure of a patent foramen ovale, guided by transesophageal echocardiography.

**Results:**

We have performed this surgery in 9 patients. None of them had a previously diagnosed patent foramen ovale. Mean age was 74 (±5) years, Operation time was 175 min (± 34 min), Clamp time 35 min (± 16 min) and Cardiopulmonary bypass time 80 (±17 min). Mortality was 0%. Periprocedural transesophageal echocardiography revealed closure of the patent foramen ovale in all cases.

**Conclusion:**

We report a new surgical method for transoesophageal echocardiography controlled closure of a patent foramen ovale without the need for an atriotomy. This new technique is especially useful for the closure of patent foramen ovale in the setting of on-pump and off-pump coronary artery bypass graft surgeries alike.

## Introduction

The patent foramen ovale (PFO) is present in approximately 27% of the general population [1]. With the widespread use of transesophageal echocardiography (TEE) during cardiac surgery, more PFO are diagnosed intraoperatively [2]. There are so far no general recommendations, on how to treat a PFO diagnosed intraoperatively. The traditional surgical technique would be to perform a direct closure via an atriotomy. This, of course, makes on pump surgery and double venous cannulation mandatory. Leaving the PFO intact however, might expose the patient to unclear immediate and long-term risks (eg, hypoxemia and paradoxical embolism such as stroke). So far, according to a survey among cardiac surgeons in the United States, only approximately 28% of surgeons always close a PFO during cardiac surgery, even if off-pump-surgery was initially planned [3].

This low rate of closure of an incidentally found PFO during cardiac surgery is most likely explained by the fact, that the “classical” approach to PFO closure involves cardiopulmonary bypass with bicaval cannulation and is therefore a major change in the planned procedure.

Hence, we developed an easy surgical method for TEE guided closure of a PFO transatrially without the need for an atriotomy or double venous cannulation. This new technique can be performed as well on and off pump, making it a feasible approach for eligible patients.

## Methods

### Surgical technique

**Fig. 1:**
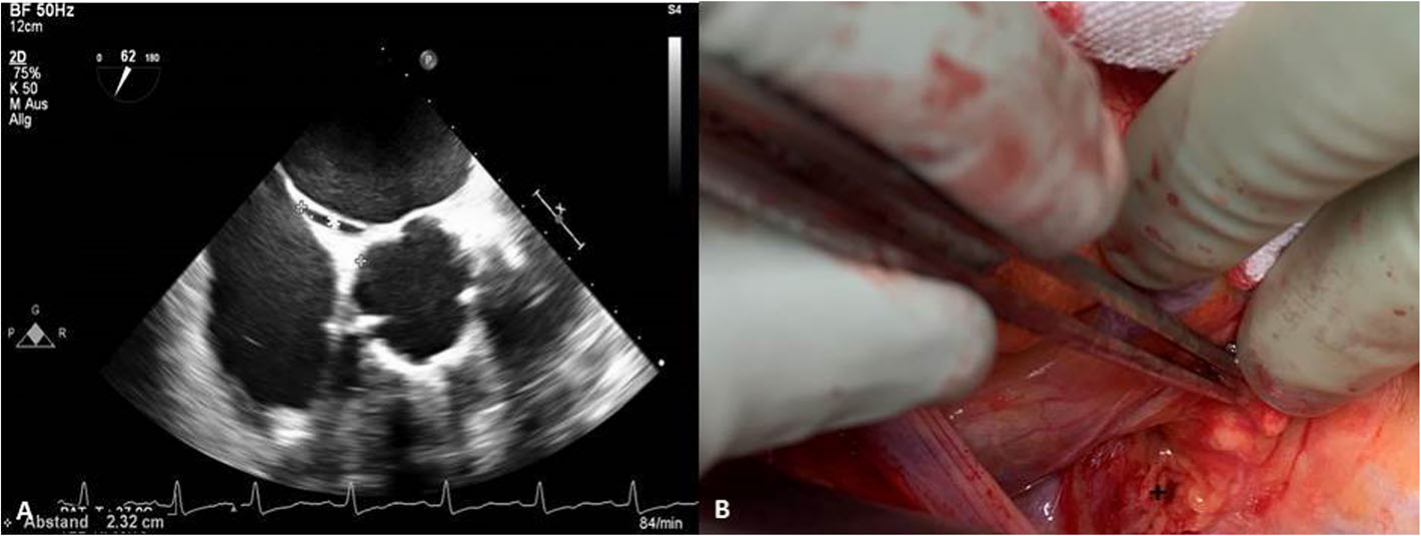
**a**: TEE of the patent foramen ovale pre-operativly (*). **b**, preparation of the interatrial grove prepared for the transmyocardial closure (+)


We retrospectively analyzed all patients that received the transatrial PFO closure procedure at our institution from 2018 to 2019. The surgeries were performed by three cardiac surgeons. In total 9 initial patients were included in our study. The planned surgery in our cohort included aortic valve replacement, coronary artery bypass grafting (CABG) (both On- and Off –pump) and combination of CABG and aortic valve replacement (See Table 1). Patients gave consent to PFO closure if incidentally detected during the operation. For a stepwise instruction, please refer to Fig. 1 and Video 1. After full sternotomy, dissection of the interatrial groove (Sondergraads / Watersons) in front of the right superior pulmonary vein was performed; enfolding the tissue between the right superior pulmonary vein and the venous sinus of the atrium (these walls join to form the septum secundum). Under TEE guidance (Phillips Curewave Affiniti 70G, Probe: X72T, Philips Healthcare, Andover, Massachusetts, USA) probing using blunt instruments was performed to localize the PFO. Depending on TEE and the distance from the orifice of the superior vena cava (SVC) to the closest aspect of the PFO, two 3/0 Prolene (SH) horizontal mattress sutures supported by teflon felts were passed from the left atrium to the right atrium to close the PFO. The suture margins to the PFO should be at least 3–4 mm in length. Mitral-and tricuspid valve function was assessed and the sutures were controlled for bleeding. Subsequently, TEE controlled bubble testing was performed to assure successful closure of the PFO. Subsequently, the planned surgery was performed.

**Table 1:** Patient’s collective

total	*n* = 9
Gender male	6 (67)
Age	74 (±4.9)
Coronary artery disease	6 (75)
Aortic valve stenosis	4 (50)
Euroscore	2.04 (±0.67)

Patient’s collective, Continuous variables are presented with the standard deviation; categorical variables are presented as number (%)

**Additional file 1 Video 1**. Part 1: Transesophageal echocardiography peri-operative bubble testing resulting in significant shunting between the right and left atria. Part 2: Operative technique using transmyocardial, pledgeted sutures to close the paten foramen ovale. Part 3: Postoperative transesophageal echocardiography showing the complete closure of the patent foramen ovale with subsequently negative bubble test.

### Techniques for transesophageal echocardiography

**Fig. 2:**
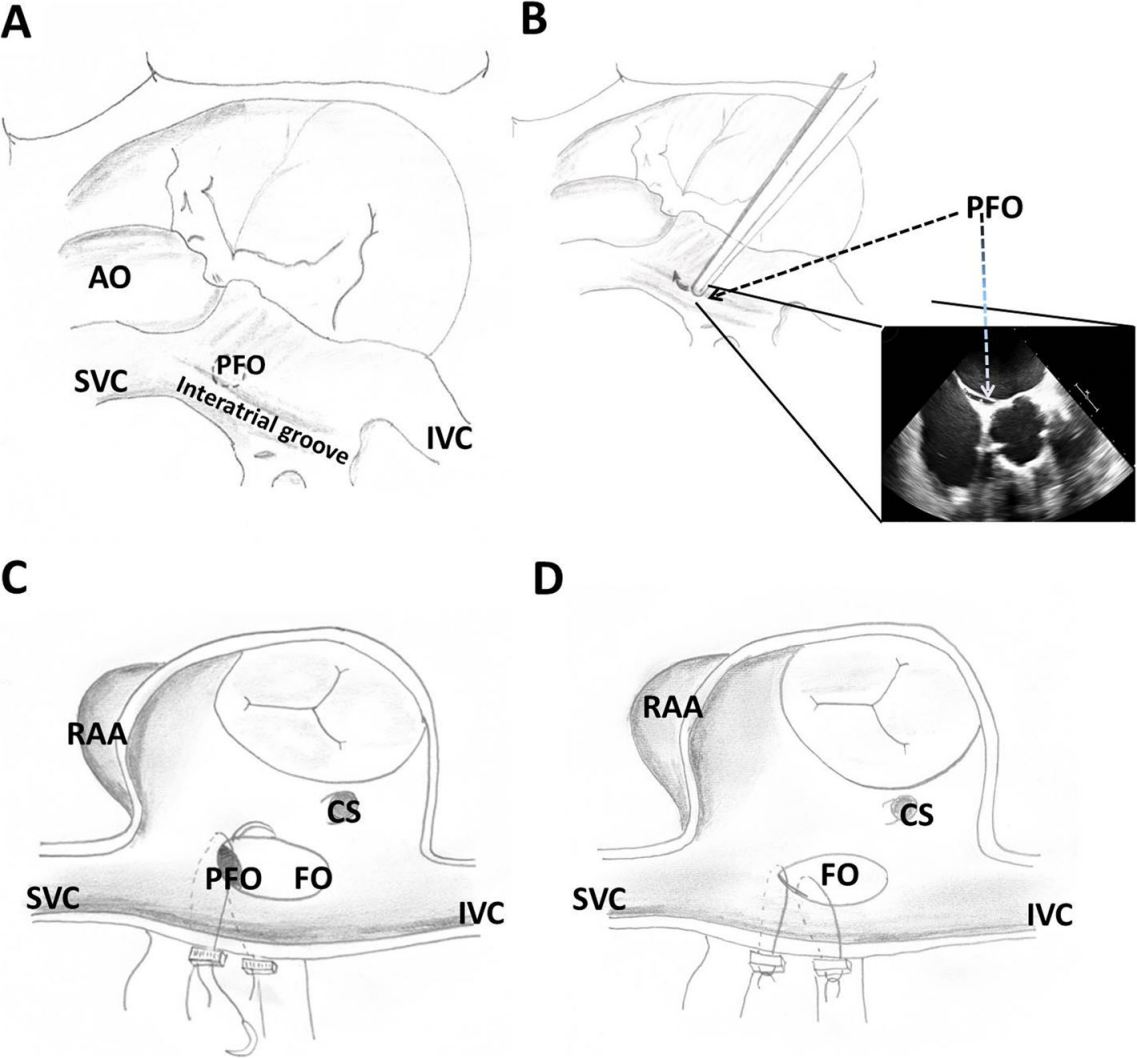
The operational procedure in detail. **a**: Identification of the interatrial groove with subsequent removal of fat tissue. The PFO is usually slightly above the groove towards the superior vena cava **b**: “Probing” using forceps under transesophageal echocardiographic guidance to identify the exact location and size of the PFO **c**: Two 3/0 Prolene horizontal mattress sutures supported by teflon felts were passed from the left atrium to the right atrium to close the PFO. The margin between the PFO and the suture should be 3–4 mm. **d**: The sutures are carefully tightened and the PFO is subsequently closed. If necessary, further sutures can be applied until safe closure of the PFO. (SVC: superior vena cava, IVC: inferior vena cava, AO: aorta, PFO: patent foramen ovale, FO: Fossa ovalis, RAA. Right atrial appendage)

A standard procedure in perioperative TEE is to exclude the prevalence of PFO by using color doppler in addition to a provocative maneuver like Vasalva and agitated colloidal fluid as contrast injection in a bicaval view. After detecting a PFO the optimal view for the most prominent appearance of the PFO has to be found (Fig. 2a). Therefore the ordinary bicaval view had to be adjusted by different rotation and slight left and right turns of the probe. To guide the surgeon to the opening of the PFO, forceps induced slight impressions (“Probing”) were visualized (Fig. 1b). Although especially for less experienced examiners the transfer of the anatomical correct direction from a 2D-bicaval-view on the screen to the real life image of the surgeon might be difficult, this procedure is relatively easy to learn and to apply.

## Results

We have performed this surgery in 9 patients. None of them had a previously diagnosed PFO. Patients gave written consent to include possible alterations to the planned procedure. Five patients received CABG, one of them as a re-operation, two patients received a biological aortic valve replacement and two patients received aortic valve replacement and concomitant CABG. Mean age was 74 (±5) years, Operation time was 175 min (± 34 min), Clamp time 35 min (± 16 min) and Cardiopulmonary bypass time 80 (±17 min) (See Table 2). PFO were relatively small in size (0.21–0.42 cm) (See Table 3). There were no periprocedural hypoxias. There were no peri- or post-procedural complications except one patient with prolonged weaning due to allergic asthma/delir, and one re-thoracotomy due to bleeding, independently from the PFO closure. ICU stay was 1.6 (±0.6) days. Mortality was 0%. Periprocedural TEE revealed closure of the PFO in all cases. None of the patients revealed any new valvular pathologies after the PFO closure.

**Table 2:** Operative data

Operation time (min)	175 (±34)
CPB time (min)	80 (±17)
clamp time (min)	35 (±16)
OPCAB Procedure	1(11.1)
AVR	4 (44.4)
CABG	7 (77.8)
Rethoracotomy for bleeding	1 (11.1)
Mortality	0 (0.0)
Postoperative Apoplex	0 (0.0)

Operative Data, Continuous variables are presented with the standard deviation; categorical variables are presented as number (%)

**Table 3:** Operative details/postoperative course

Patient Nr.	Operational procedure	Diameter of PFO	Max. catecholamines	complications	Hospital length of stay
1	CABG (LIMA-LAD, RA (as T-graft) to PLA1-PLA2; PFO closure	0.21 cm	No postoperative catecholamines	none	10 days
2	LIMA-LAD as redo CABG; PFO closure	0.22 cm	max. 0.147 μg/kg/min Norepinephrine max. 1.83 μg/kg/min Dobutamine	postoperative delir and asthma	12 days
3	CABG (LIMA-LAD, ACVB-PLA-RIVP); PFO closure	0.32 cm	No postoperative catecholamines	Postoperative DDD pacemaker implantation	13 days
4	AVR, LIMA-LAD; PFO closure	0.42 cm	No postoperative catecholamines	none	12 days
5	AVR, PFO closure	0.32 cm	Max. 0.127 μg/kg/min norepinephrine	none	17 days
6	AVR, CABG (LIMA-LAD, ACVB-PLA-RIVP, PFO closure	n.a. cm	Max. 0.152 μg/kg/min norepinephrine	none Postoperative re-sternotomy due to bleeding	14 days
7	AVR, PFO closure	0.22 cm	max. 0.157 μg/kg/min Norepinephrine max. 7.37 μg/kg/min Dobutamine	none	17 days
8	CABG RA (as T-graft) to PLA1-RIVP; PFO closure	0.24 cm	No postoperative catecholamines	none	8 days
9	CABG (LIMA-LAD, RA (as T-graft) to PLA1 OPCAB, PFO closure	0.25 cm	max. 0.167 μg/kg/min Norepinephrine	none	7 days

Operative details of patients, including the postoperative course (CABG: Coronary artery bypass grafting, *RA* radial artery, *LAD* left anterior descending, *PLA* posterolateral branch, *OPCAB* Off-pump coronary artery bypass grafting, *AVR* aortic valve replacement)

## Discussion

The management of incidental PFO in cardiac surgery has been reviewed and extensively debated in the literature [4–7]. So far, the indication on whether to close a PFO remains on the 3 categories [3]: Category I, close in high-risk settings; category II, close in surgical procedures that involve an atriotomy; and category III, consider on a case-by-case basis at the surgeons discretion.

Category I patients with a high risk include left ventricular assist device patients and heart transplant recipients. These patients are at a high risk hypoxemia with right-to-left shunting through a patent PFO. Category II patients are undergoing procedures that involve bicaval cannulation and/or atriotomy. Closure of the PFO in these operations mean minimal deviation from the surgical plan and general consensus is therefore to close PFO in such a setting. Common operations in this category would be mitral valve procedures, tricuspid valve procedures, atrial mass resections and alike. Probably the most debated patients are in category III: incidental PFO finding during cardiac surgery such as CABG on and off pump. Dealing with a PFO in these patients can mean a major deviation from the surgical plan with the need for bicaval cannulation or even switching from off- to on-pump surgery. Especially OPCAB patients are at risk for perioperative hypoxemia due to right-to-left shunting during the mandatory heart manipulation during the bypass grafting. However, whether Category III patients should receive PFO closure is still under debate.

So far, it is generally agreed that surgical closure in these patients (Category II) might even increase the risk of perioperative stroke with no effect on survival in the long term. Therefore, the authors concluded to discourage form the closure of an incidentally detected PFO in these patients [3]. Others concluded that PFO closure may be considered in selected cases [5, 6].

Therefore, our new surgical technique omitting the need for bicaval venous cannulation and atriotomy adds to the armament of the cardiac surgeon and enables to perform off- and on-pump surgery (without bicaval venous cannulation) while still closing the PFO. The other beneficial effect is that without atriotomy, the incidence of air embolism during the PFO closure is excluded.

There are some limitations to our technique that have to be taken into account. TEE by an experienced anesthesiologist has to be available. Although we do not have the data yet, we cannot recommend our approach in patients with septal aneurysm or true atrial septum defects. The PFO should not be large and the opening of the PFO has to be identified clearly. The PFOs that were closed were relatively small in our cohort (0.21–0.42 cm) and we recommend to limit the operation to PFO sizes of PFO < 0.5 cm, since otherwise adjacent structures (tricuspid valve, coronary sinus) could be damaged. Further studies testing for closure rates and possible complications have to follow, with larger patients cohorts. Until this approach is to be proven safe with high closure rates, we recommend it for patients with low risk for paradox embolism (Category III). Patients with previous apoplexy, septal aneurysms or large PFO/ atrial septum defects should still be operated the classical approach using bicaval cannulation, atriotomy and either direct closure or if needed, patch plasty. As soon as our technique has proven reliable in closure rates, we would highly recommend this technique for VAD patients, since these already very much impaired patients with high periprocedural risk would benefit most from a safe and less invasive technique for PFO closure. However, these patients develop the highest pressure gradients and therefore a most secure PFO closure is indispensable.

## Conclusion

In conclusion, we hereby introduce our new technique for PFO closure without atriotomy and without the need for cardiopulmonary bypass. None the less, our technique has to be put to the test, preferably in a randomized, controlled trial against the two alternatives: no PFO closure and PFO closure the “classical” way.

## Data Availability

Not applicable.

## Abbreviations

PFO: Patent foramen ovale
TEE: Transesophageal echocardiography
CABG: Coronary artery bypass grafting
